# Living well while providing support: validation of LTCQ-Carer for assessing informal carers’ quality of life

**DOI:** 10.1007/s11136-023-03485-z

**Published:** 2023-08-02

**Authors:** Caroline M. Potter, Michele Peters, Maureen Cundell, Rupert McShane, Ray Fitzpatrick

**Affiliations:** 1 Health Services Research Unit, Nuffield Department of Population Health, University of Oxford, Oxford, UK; 2 Applied Research Collaboration Oxford and Thames Valley (NIHR ARC OxTV), National Institute for Health and Care Research, Oxford Health NHS Foundation Trust, Oxford, UK; 3 Oxford Institute of Population Ageing, University of Oxford, Oxford, UK; 4 Oxford Health NHS Foundation Trust, Oxford, UK

**Keywords:** Informal carer, Family caregiver, Quality-of-life scale, Carer-reported outcome measure, Mild cognitive impairment (MCI), Dementia

## Abstract

**Purpose:**

Despite international policies to support the health and wellbeing of informal (family) caregivers, there is no consensus on how to evaluate the effectiveness of carer support. We aimed to develop and validate a new quality-of-life measure for carers (LTCQ-Carer) and to assess its potential for use within a clinical pathway.

**Methods:**

Psychometric properties of LTCQ-Carer were tested through cognitive interviews (qualitative phase) and a pilot survey (quantitative phase). Participants were family caregivers of people recently diagnosed with mild cognitive impairment (MCI) or dementia, recruited through one of 14 memory clinics in south-east England. They self-completed the new measure and comparative existing measures (EQ-5D, ASCOT-Carer). Ongoing feedback from memory clinic staff on potential use of LTCQ-Carer was collected.

**Results:**

Interview participants (*n* = 10) found all draft items of LTCQ-Carer relevant and prompted inclusion of a new item on ‘time to yourself’. Responses from survey participants (*n* = 107) indicated acceptability (low missing data), high internal reliability (Cronbach’s α = 0.95), and a general construct (single factor loadings 0.43–0.86 for all items). Observation of predicted associations with EQ-5D and ASCOT-Carer supported construct validity. Responsiveness requires further testing as evidence was inconclusive. Clinical staff feedback on potential use was positive.

**Conclusion:**

LTCQ-Carer is a valid new measure for assessing family caregivers’ quality of life across broad health and social care domains, expanding the range of high-quality tools for evaluating carer support. When used concurrently with patient assessment, it could highlight carer needs and prompt appropriate family support at the earliest point in the clinical pathway.

## Plain English summary

Informal (unpaid) caregivers provide a substantial amount of support to friends and family members living with long-term health conditions such as dementia. Governments and advocacy groups around the world recognize that giving support to a loved one can impact the health and wellbeing of carers, who might need support themselves to live as well as possible. Currently there is not a consistent way to monitor the quality of life of informal carers, but clinical appointments for their cared-for relatives are opportunities to identify carers’ needs and to offer support at early stages of the journey. In this study we developed and tested a new questionnaire, the Long-Term Conditions Questionnaire for Carers (LTCQ-Carer), with informal carers whose family members had a diagnosis of mild cognitive impairment or dementia. Through interviews and then a survey, we found that the questions in LTCQ-Carer were relevant for this group of carers and covered a range of issues that could prompt support from health or social care services. Memory clinic staff found the content of LTCQ-Carer useful for starting early conversations about carers’ needs, and they were willing to use it as part of the patients’ assessment visit. Future research should test LTCQ-Carer in larger groups of people with a wide range of caregiving experiences, for use in supporting families affected by all types of long-term health conditions.

## Introduction

In this article we present the development and initial validation of a new outcome measure for informal (unpaid) caregivers to self-report their own care-related quality of life (QoL), in the context of providing support to a friend or family member with at least one long-term health condition (LTC). This work evolved from a linked study to test a new patient-reported outcome measure, the Long-Term Conditions Questionnaire, for use by people diagnosed with mild cognitive impairment (MCI) or dementia [1]. Our clinical research partners working in memory clinics noted that their patients are almost always accompanied by someone who supports them, and while assessment appointments primarily focus on the needs of the cared-for (patient), the clinical encounter provides a key opportunity for healthcare professionals to identify carers’ needs as well. Supporting unpaid carers has both health and economic implications; it is estimated that they account for 40% of the care provided for people living with dementia, equivalent to more than 40 million full-time workers globally [2].

Within England, carers’ rights to maintain their own wellbeing [3, 4] and the high personal costs they bear [5, 6] are widely recognized. The health and wellbeing impacts of responding to the changing needs of a person with dementia [7] and managing a wider web of support [8] can be substantial. The need for effective interventions to support carers, particularly in the context of dementia, has been recognized for decades [9]. A recent clinical editorial [10] renewed the call for effective interventions for people supporting older adults, highlighting cultural variation in carers’ experiences [11] and dynamics within multi-carer networks that include informal caregivers living distantly from family [12].

In spite of expanding national and international policy to support carers’ QoL and wellbeing [13–17], evidence on how these outcomes can best be achieved and assessed is inconsistent [18–20]. Clinical guidelines for supporting carers are still emerging [21], and there is currently no consensus approach for evaluating the effectiveness of carer support. In their review of quality-of-life measures for carers of people with dementia, Manthorpe and Bowling [19] note that there are relatively few measures for carers to report their own health status and wellbeing, compared to proxy scales for carers to report on behalf of the cared-for. Carer-focused measures that have been developed more recently include the Carer Experience Scale [22], CarerQoL [23], and ASCOT-Carer [24]; all of these are designed as generic measures of care-related QoL primarily for use in economic evaluations and/or social care settings. Similarly to generic measures for health-related quality of life such as the EQ-5D [25], these measures are valuable tools for comparative analyses but might have limited application in clinical settings, where mitigating the wider impacts of long-term health conditions on informal carers could be addressed alongside impacts on patients.

In response to clear need for high-quality tools to assess carer QoL across a range of carer experiences [15, 17], we hypothesized that content from the Long-Term Conditions Questionnaire (LTCQ) could be adapted to capture outcomes of importance for people supporting friends or family members with a long-term health condition. LTCQ is a self-administered questionnaire designed to capture a holistic construct of ‘living well with LTCs’, potentially complementing symptom-burden assessments through disease-specific measures. LTCQ was developed through best-practice methods including literature reviews, stakeholder and public consultation [26], qualitative interviews with patients [27], pre-testing alongside a translatability assessment [28], and a validation survey among a large sample of health and social care users in England [29]. Rasch analysis of item performance [30] and development of a mapping algorithm for calculating health utilities values from LTCQ responses [31] have further increased the measure’s robustness. The 20-item LTCQ captures a broad range of domains including: sense of control, ability to do meaningful activities, safety inside and outside the home, burden of treatments and services, negative experiences including loneliness and stigma, confidence to self-manage LTCs, and ability to live life as one wants. Collectively these studies suggest LTCQ’s relevance for use by diverse populations with wide-ranging experience of LTCs.

The core concepts underpinning LTCQ’s theoretical framework [27] are the broader functional and social impacts of living with long-term conditions, experiences of navigating (potentially complex) services and support, and experiences of self-managing LTCs outside of formal care settings. The resulting domains are potentially relevant for, but not specific to, patients and carers experiencing the impacts of memory problems. In designing this study the project team considered use of existing and emerging condition-specific measures (e.g. C-DEMQOL and SIDECAR, which were in development at the same time), but the conceptual breadth of LTCQ was preferred by clinical partners, and LTCQ’s developers saw potential for its use as a common metric across different specialist services. In adapting LTCQ’s content for use by informal carers, we build on previous work highlighting the critical but often overlooked impact of chronic illness on family members [32]. LTCQ-Carer is intended to measure care-related quality of life with reference to the broader health- and social-care related domains captured by LTCQ, which are affected by the presence of one or more LTC in the cared-for person. In this study, we aimed to understand if LTCQ-Carer provides a valid means of assessing care-related quality of life, and if use of LTCQ-Carer could fit into an existing clinical pathway such that carers’ needs could be identified and supported concurrently with patients’ needs.

## Methods

LTCQ-Carer was developed through cognitive interviews on draft questionnaire content with carers of people living with mild cognitive impairment (MCI) or dementia. Original phrasing of LTCQ items was retained as far as possible, but with statements made in relation to ‘providing care and support’ rather than ‘your health condition(s)’. The research team reviewed each item for meaning, with some items adapted in order to make more sense from a carer perspective (e.g. ‘felt as independent as you wanted’ for carers versus ‘felt more dependent on others than you would like’ for patients). Following COSMIN guidelines [33], psychometric properties of LTCQ-Carer were then tested through a two-phase mixed methods design: qualitative research via cognitive interviews with carers (Phase 1) to test content validity and appropriateness, followed by quantitative assessment of a pilot survey (Phase 2) to test acceptability, structural validity, construct validity, and responsiveness.

### Participant recruitment and data collection

Participants were recruited following a diagnosis in their cared-for person of either MCI or dementia. Diagnosis of the patient and regular support from an informal carer were confirmed by clinical staff during assessment at one of 14 memory clinics based within two National Health Service (NHS) Trusts in South East England. During the patient’s assessment, carers had a separate conversation with memory clinic staff and were asked about their willingness to participate in research. Those who met the inclusion criteria (confirmed diagnosis in the cared-for person, willing and able to participate, at least 18 years of age, able to communicate in English) were given a study pack that included an invitation letter and participant information sheet.

For Phase 1, cognitive (‘think aloud’) interviews [34] were conducted by the lead researcher to assess the comprehensibility and content of adapted LTCQ items from the carer’s perspective. Participants completed the draft LTCQ-Carer measure in the interviewer’s presence and were prompted to discuss their choices when selecting item responses. An interview guide structured discussion around meaning of specific concepts and terms within the items, clarity of the instructions for completing LTCQ-Carer, suitability of the response options available, whether there were any unclear or unsuitable questions, whether any important concepts were missing, and whether or not participants would find it useful to complete LTCQ-Carer in health or social care settings. Interviews were conducted at the carer’s home and included some open-ended discussion of participants’ experiences of caring and their own health. Interviews were held in two rounds (minimum five participants per round), with review and potential modification of the questionnaire after each round in response to participant feedback.

Phase 2 of the study consisted of self-administered surveys returned by post, taken at two time points four months apart. Participants were again recruited through memory clinics using the same inclusion criteria outlined above. The study packs included the full survey (Survey 1), which was comprised of the LTCQ-Carer (questions 1–21), a comparative generic measure for health-related quality of life (EuroQol five-dimensional descriptive system with visual analogue scale: EQ-5D-5L with EQ VAS, questions 22–27) [25], relationship with cared-for person (question 28), a comorbidity scale (question 29), a comparative measure for social-care-related quality of life (ASCOT-Carer, questions 30–36) [24], demographics (questions 37–41), and a box for free-text comments. Participants who were willing to take part in a follow-up survey provided their contact details and were sent a shorter questionnaire (Survey 2) four months later, spanning the period of initial post-diagnosis support provided by memory clinics that typically lasts three months (after which patients are discharged back to primary care and/or ongoing community-based support). The follow-up survey included LTCQ-Carer (questions 1–21), a question on change in health status since the previous survey, a question on health service use in relation to the cared-for person’s memory problems, and demographics (questions 24–26).

To assess the potential for use of LTCQ-Carer within this aspect of the clinical pathway (i.e. MCI or dementia diagnosis through memory clinics), the lead researcher maintained contact with memory clinic staff throughout all phases of the study, for ongoing informal feedback on study recruitment and potential barriers/facilitators for routine collection of carer-reported outcomes data.

### Data analysis

Data analysis followed a similar protocol as for the linked study with memory clinic patients, previously described [1]. Briefly, Phase 1 qualitative data were analysed using a framework reflecting the interview guide, which was used to structure a data extraction template used during the interviews and during subsequent play-back of the audio recordings. For each round of cognitive interviews, participant’s comments on each item of the LTCQ and on the broader topics (e.g. clarity of instructions, appropriateness of response options) were collated and discussed amongst the research team. Comments were categorised to highlight possible amendments needed to questionnaire items: green (no concerns), orange (requires discussion/clarification), or red (problems raised, may require amendment). Cognitive interviews were conducted until no further areas for potential amendment were identified, with a record kept of the research team’s response to each query or comment raised by participants.

Phase 2 survey data were entered into SPSS (version 27), a statistical software package. LTCQ-Carer items were scored on a scale from 0 (most negative response) to 4 (most positive response). For responses for which at least 19 LTCQ-Carer items (90% of questionnaire) had been answered, the sum of item scores was calibrated to give an overall LTCQ-Carer score ranging from 0 to 100. EQ-5D-5L index values were calculated from a value set for England [35]. EQ VAS scores did not require further transformation from their reported range of 0 to 100. ASCOT-Carer was calculated as a sum score of items ranging from 0 to 21 [24]. Spearman’s rank correlation coefficient was calculated to test the associations between LTCQ-Carer score and EQ-5D-5L index, EQ VAS score, and ASCOT-Carer score.

For Survey 1, all LTCQ-Carer items were examined for missing data, and the measure as a whole was examined for floor/ceiling effects (i.e. 15% or more of respondents scoring the lowest/highest possible score). Internal consistency (i.e. extent to which items correlate with each other, implying a common underlying construct) was assessed with Cronbach’s alpha statistic [36], with good internal consistency interpreted as α of 0.7 or higher. Exploratory factor analysis of the 21 LTCQ-Carer items was undertaken, using parallel analysis [37] to guide retention of factors and 0.3 as the minimum threshold for factor loadings. The appropriateness of scoring items as a single scale was evaluated through examination of inter-item correlations (acceptable if 0.8 or less) and item-total correlations (acceptable if 0.3 or more) [38]. One-way analysis of variance (ANOVA) was employed to compare distributions of LTCQ-Carer scores among sub-groups within the sample (e.g. demographics, health conditions reported) as a test of known-groups validity, with carers of older age and higher numbers of their own LTCs hypothesized to have lower care-related QoL.

Survey 2 responses were also analysed for levels of missing data, floor/ceiling effects, and internal consistency. Changes in LTCQ-Carer scores were calculated for each respondent between the two survey time points. Changes in LTCQ-Carer score were analysed using the paired t-test statistic, with the aim of comparing groups reporting improvement, no change, or decline in global health status across the survey time points (recorded as a single item on overall health change in Survey 2). Change for responsiveness analysis was defined as a statistically significant increase or decrease in individual-level (paired) LTCQ-Carer scores among these defined sub-groups, with LTCQ-Carer scores predicted to increase for those reporting improvement since Survey 1 and LTCQ-Carer scores predicted to decrease for those reporting decline.

## Results

### Phase 1: cognitive interviews to develop questionnaire content

Ten interviews were conducted across two rounds. Seven were with the partner/spouse who lived with the memory clinic patient, one was with an adult child who lived with the patient, and two were with adult children who provided regular support but did not live with the patient. The patient was present during three interviews, which in two cases meant some reluctance on the part of the carer to elaborate on their responses regarding the full impact of their caring responsibilities (communicated informally to the interviewer when the patient was not within hearing distance). All carers were able to complete the draft questionnaire independently in less than 8 min, with an average completion time of 5 min. No concerns were raised about the format or readability of the questionnaire, which was completed in full by all participants.

Table 1 summarises the feedback given by interview participants, with amendments considered or made to the questionnaire in response. Across all participants, all questionnaire items were found to be relevant. Some items were amended to better clarify the concept of interest (e.g. Question 6 on material resources needed to support caring, Question 12 on experiences of social stigma). An additional item on ‘enough time to yourself’ was added after the first round of interviews; subsequent interview participants found this item relevant, so it was retained for the survey phase in which LTCQ-Carer was tested with 21 items. Minor wording or formatting changes were made for clarity, for example clearer labelling and emphasis through bold font of the ‘Not applicable’ response options for Questions 13 and 14 (for respondents who had not received any support services or treatments for their cared-for person in the defined four-week recall period; these item responses were scored as 4—highest score indicating no problems reported—for comparability). Issues raised in the second round of interviews were minor formatting suggestions that resulted in no further changes to the draft questionnaire.

**Table 1:** Interview phase participants and results

Participant	Gender	Relationship	Patient’s LTCs	Carer’s LTCs	Key points raised	Response
1	Male	Partner living with patient	Alzheimer’s, bowel problems, blood pressure management, worsening eyesight	(no LTCs mentioned)	Felt questions would be more relevant as illness progressed; answered easily to indicate minimal impact on himself at the moment. Wanted N/A response option for Q13, which he found least relevant (interpreted ‘services’ as social care)	For Question 13, ‘Not applicable’ added to response option for no services used in past four weeks
2	Male	Adult child, living at different address to patient (same town)	Dementia/Alzheimer’s	(no LTCs mentioned)	Suggested content on impact of relationship with patient. Expand examples of ‘everything you need’ for Q6. Noted that his mother (primary carer) would have answered questions differently and that his responses might change over time. Saw value in repeat measures, if questionnaire responses led to action/support	Amended Question 6 to make the wording more generalised, prompted for meaning in round 2 interviews
3	Female	Spouse living with patient	Mild cognitive impairment, diabetes	(no LTCs mentioned)	Thought the questionnaire would show change as patient’s memory problems worsened. Valuable if it prompts support and action. Found Q12 hard to interpret	Amended Question 12 to wording suggested by participant
4	Male	Adult child living with patient	Dementia/Alzheimer’s	(no LTCs mentioned)	No missing content. Q13 hardest to answer (unsure what ‘services’ covered). Q6 need to refer to more than ‘equipment’. Value of questionnaire in prompting discussion of issues that carers might be reluctant to raise or that might change over time. But in the absence of feedback it could be frustrating to complete	Amended Question 6 to make the wording more generalised, prompted for meaning in round 2 interviews
5	Female	Partner living with patient	Memory problems (diagnosis not specified), heart condition, kidney problems, knee problems	Emphysema	Suggested additional question on getting enough time to yourself. Suggested expanding response options to include ‘usually’. Thought questionnaire would be more relevant to people with more caring responsibilities and could show changes over time	Added new item on having enough time to yourself
6	Female	Spouse living with patient	Dementia, heart condition	Chronic pain, depression, hip replacement	Being asked the questions ‘takes a bit off your shoulders’ because ‘I don’t talk to everyone like this’. Q16 most difficult to answer because response options do not capture changes in knowledge/skills over time	After discussion, no changes made (changes in knowledge would be captured by repeat measures)
7	Male	Spouse living with patient	Memory problems (diagnosis not specified)	(no LTCs mentioned)	Generalised but relevant content. Suggested splitting ‘coping’ into physical and emotional domains. Useful for monitoring carer’s well-being over time, and thought that responses could reflect changes in carer’s health status, but needs to be balanced with burden of frequent questionnaire completion	After discussion, no changes made (no problems with item completion; consistency with validated LTCQ item)
8	Female	Spouse living with patient	Alzheimer’s/dementia, Parkinson’s, COPD, depression	angina, Morton’s neuroma (nerve pain in feet affecting mobility), diabetes	Valued questionnaire as a monitoring tool and would want access to responses to self-monitor. Some questions could be difficult to answer over a 4-week timeframe because of daily changes in own health. Difficult to distinguish the impact of caring versus health generally for question on physical activity. Preferred ‘most of the time’ to ‘often’ as a response option	After discussion, no changes made (no problems using current response options; consistency with LTCQ response options)
9	Female	Spouse living with patient	Short-term memory loss, low mood, bladder cancer, gall stones	(no LTCs mentioned)	‘Nice and easy’ to complete as she saw husband’s condition as mild and did not self-identify as a ‘carer’. Saw value in filling out questionnaire as part of memory clinic assessment, but would be put off by a lack of direct feedback from staff	(No suggestions for changes)
10	Female	Adult child, living at different address to patient (in walking distance)	Dementia, previous heart attacks	(no LTCs mentioned)	All items relevant, but noted health impacts (Q10) could be different for physical versus mental health. Felt that confidence to manage would change with mother’s condition deteriorating. Main concern was that caring was framed entirely as burden, with no questions on positive impacts	Amended Q12 wording for consistency with other items

### Phase 2: pilot survey for initial validation of LTCQ-Carer

For Survey 1, a response rate of 27% (109/410) was achieved. Two repeat surveys from participants already recorded were excluded, for an analysis sample of *n* = 107. Most respondents (75%) provided contact details to take part in the four-month follow-up, with a response rate of 69% (51/74) achieved for Survey 2.

Respondents for Survey 1 had a mean age of 67 years (range 41–90 years), were 63% female, were 93% white British, and were 87% married or in a civil partnership. The majority of respondents (68%) described their caring relationship as ‘family member who lives with them’, with a notable minority (26%) describing themselves as ‘family member who lives at a different address’ than the cared-for. Most respondents (79%) completed the questionnaire independently, while 18% reported giving their own answers but having some help in physically completing the form. A majority of respondents (57%) reported living with at least one long-term health condition themselves; the most frequently reported conditions were arthritis (27%), high blood pressure (25%), depression (9%), heart disease (7%), and lung disease (7%). Around 25% of the sample experienced multiple health conditions, including four respondents who lived with at least four different LTCs.

### Acceptability, internal consistency, and structural validity

Item-level response frequencies and correlations with the overall LTCQ-Carer score are shown in Table 2**.** All items were below the 10% missing data threshold where bias in statistical analysis becomes a concern [39], with most items having 0–3% missing responses. As observed for the original LTCQ measure, responses skewed towards the most positive response options for some items. This was most pronounced for items 7 (felt safe inside the home), 8 (felt safe outside the home), 13 (never found services difficult to cope with), and 14 (never found treatments difficult to cope with), for which more than half of respondents selected the most positive response option. As for LTCQ, these items were considered acceptable to retain, given that selection of any of the more negative responses for these items would give a clear signal of potential support needs.

**Table 2:** LTCQ-Carer item-level responses including missing data, item-total correlations, and factor loadings

	Percent for each item response option						Item-total correlations	Factor loadings^b^
LTCQ-Carer item	Never	Rarely	Sometimes	Often	Always	(missing)		
1. Felt able to cope well with giving care and support	0	0	24.3	32.7	42.1	0.9	0.73	0.76
2. Felt able to fulfil other responsibilities	0	6.5	22.4	31.8	39.3		0.81	0.84
3. Able to be as physically active as you want	4.7	13.1	20.6	21.5	40.2		0.80	0.83
4. Felt in control of daily life	4.7	8.4	20.6	27.1	39.3		0.81	0.84
5. Able to take part in enjoyable activities	6.5	9.3	29.9	15.9	38.3		0.78	0.80
6. Have everything you need when giving support	0.9	7.5	30.8	18.7	41.1	0.9	0.69	0.71
7. Felt safe inside the home when giving support	0	0	7.5	18.7	73.8		0.42	0.43
8. Felt safe outside the home when giving support	1.9	0.9	7.5	24.3	64.5	0.9	0.45	0.47
9. Felt as independent as you want	3.7	9.3	29.9	23.4	33.6		0.78	0.82
10. Felt your health has been affected by giving support^a^	32.7	15.9	30.8	12.1	8.4		0.59	0.59
11. Felt lonely due to giving care and support^a^	40.2	18.7	28	8.4	4.7		0.53	0.53
12. Felt people were not understanding about your caring responsibilities^a^	25.2	24.3	31.8	13.1	4.7		0.64	0.65
13. Found services you use in relation to giving care and support difficult to cope with^a^	66.4	9.3	9.3	7.5	2.8	4.7	0.45	0.46
14. Found treatments taken by the person you care for difficult to cope with^a^	56.1	20.6	15	3.7	1.9	2.8	0.51	0.51
15. Felt giving care and support made you unhappy^a^	30.8	23.4	31.8	8.4	2.8	2.8	0.66	0.68
16. Felt you knew enough about how to give support	4.7	15.9	24.3	25.2	28	1.9	0.48	0.48
17. Had enough social contact with other people	2.8	8.4	22.4	24.3	40.2	1.9	0.70	0.72
18. Had enough help in giving care and support	3.7	12.1	26.2	21.5	29.9	6.5	0.71	0.72
19. Felt you get enough time to yourself	9.3	14	23.4	22.4	29	1.9	0.74	0.76
20. Felt confident in managing daily caring responsibilities	0.9	5.6	22.4	29	40.2	1.9	0.77	0.79
21. Felt able to live your life as you want	8.4	18.7	18.7	24.3	28	2	0.83	0.86

^a^Negatively phrased item: reverse scoring applied

^b^One-factor solution, Principle Axis Factoring (no rotation applied for single factor)

LTCQ-Carer showed high internal consistency (Cronbach’s α = 0.95) across its 21 items in this sample. The suitability of the dataset for exploratory factor analysis was confirmed through the Kaiser–Meyer–Olkin measure of sampling adequacy (0.89) and Bartlett’s test of sphericity (highly significant, *p* < 0.001). Results of parallel analysis using O’Connor’s method [37] for principal components analysis indicated retention of one factor, consistent with the original LTCQ measure in two samples [1, 25]. A one-factor solution (principal axis factoring, no rotation applied for only one extracted factor) showed that all 21 items contributed to the general factor to explain 50% of variance, with loadings ranging from 0.43 to 0.86. The item-total correlations in Table 2 further indicate that all items contribute to a general construct and can be used to calculate a composite score for LTCQ-Carer.

**Fig. 1:**
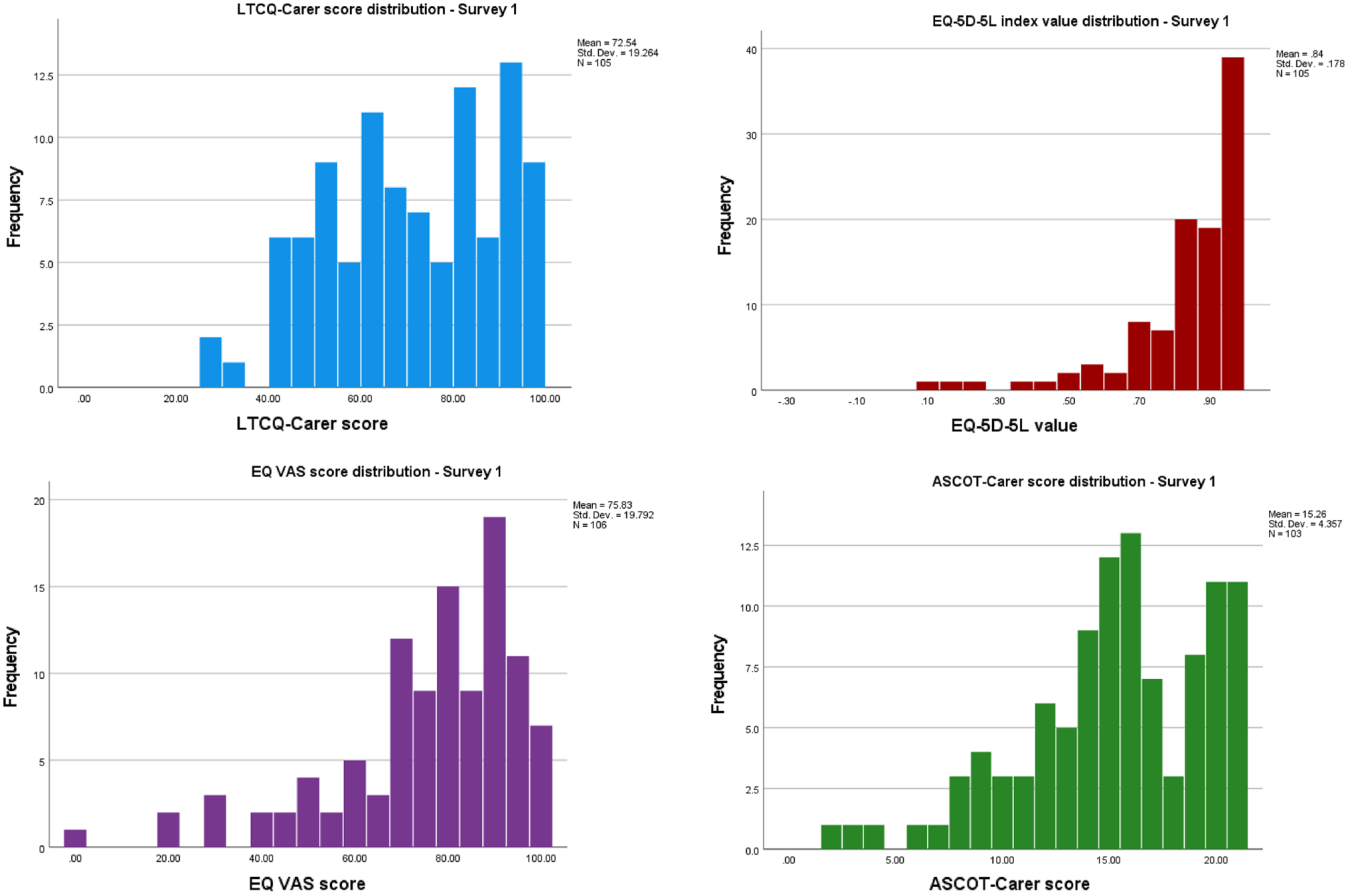
Distribution of outcome measure scores (*n* = 107)

Scores for all outcome measures, with variations by sample characteristics, are shown in Table 3. Across all measures, carers who reported living with two or more LTCs themselves had significantly lower scores than carers reporting one or zero LTCs. Lower scores on LTCQ-Carer and ASCOT-Carer were observed for carers not currently in a partnership compared to married/partnered respondents, although statistical significance in score difference was only observed for ASCOT-Carer. No differences in outcome measure scores were observed by gender, and the number of non-white respondents was too low to do a sub-group analysis by ethnicity. Comparison across age groups yielded no statistically significant differences in scores, although it was observed that mean LTCQ-Carer score was lowest in the youngest group (under 60 years) while mean EQ-5D index value was lowest in the oldest group (80 years or older). In examining distributions of scores for all outcome measures (Fig. 1), we observed that scores for the EQ-5D-5L index value were heavily skewed towards the maximum positive score in this sample, whereas other scores including LTCQ-Carer were distributed across a fuller range.

**Table 3:** Survey respondent characteristics in relation to LTCQ-Carer, EQ-5D, and ASCOT-Carer scores

Sample characteristics	N (%)	LTCQ-Carer mean score (SD, SE)	EQ-5D mean index value (SD, SE)	EQ VAS mean score (SD, SE)	ASCOT-Carer mean score (SD, SE)
Total sample	107 (100%)	72.5 (19.3, 1.9)	0.84 (0.18, 0.02)	75.8 (19.8, 1.9)	15.3 (4.4, 0.4)
		(range: 25–100)^a^	(range: 0.07–1.0)^a^	(range: 0–100)^a^	(range: 2–21)^a^
Age (range: 41–90 years)					
Under 60 years	34 (32%)	68.1 (19.4, 3.3)	0.87 (0.15, 0.02)	75.3 (18.9, 3.2)	15.5 (4.4, 0.8)
60–79 years	56 (52%)	73.8 (19.0, 2.6)	0.84 (0.17, 0.02)	77.1 (19.6, 2.6)	15.7 (3.8, 0.6)
80 years or older	17 (16%)	77.6 (19.2, 4.8)	0.76 (0.24, 0.06)	72.6 (22.9, 5.6)	15.3 (4.4, 0.9)
Gender					
Female	67 (63%)	71.6 (19.4, 2.4)	0.82 (0.18, 0.02)	74.7 (20.8, 2.6)	15.2 (4.4, 0.6)
Male	37 (35%)	73.4 (19.6, 3.2)	0.87 (0.18, 0.03)	77.7 (18.5, 3.0)	15.3 (4.4, 0.7)
Ethnicity					
White	100 (94%)	72.9 (19.4, 2.0)	0.84 (0.18, 0.02)	76.3 (19.9, 2.0)	15.4 (4.4, 0.4)
Non-white	3 (3%)	53.5 (10.4, 6.0)	0.82 (0.11, 0.07)	55.0 (13.2, 7.6)	11.3 (1.5, 0.9)
Marital status*					
Married/partnership	87 (81%)	73.3 (20.0, 2.2)	0.83 (0.18, 0.02)	75.8 (20.0, 2.2)	15.6 (4.4, 0.5)*
Not married/not in partnership	16 (15%)	65.7 (14.5, 3.6)	0.86 (0.18, 0.04)	75.1 (20.5, 5.1)	13.1 (4.1, 1.0)*
Health conditions**					
No LTCs	46 (43%)	74.5 (16.4, 2.4)	0.89 (0.11, 0.02)**	80.6 (18.4, 2.7)**	15.7 (3.6, 0.5)**
One LTC	34 (32%)	74.4 (20.7, 3.6)	0.84 (0.13, 0.02)**	77.4 (18.4, 3.1)**	16.5 (4.0, 0.7)**
2 or more LTCs	27 (25%)	66.9 (21.4, 4.1)	0.74 (0.26, 0.05)**	65.9 (20.9, 4.0)**	13.0 (5.1, 1.0)**

*SD *standard deviation, *SE* standard error of the mean

^a^Score ranges are those observed within the study sample. Theoretical ranges for each measure are 0 to100 for LTCQ-Carer, -0.285 to 1.0 for EQ-5D-5L index value, 0 to 100 for EQ VAS, and 0 to 21 for ASCOT-Carer. For all measures higher scores indicate better health/social care-related quality of life

^*^For marital status, statistically significant difference between groups in mean ASCOT-Carer scores at p < 0.05 level

^**^For number of health conditions, statistically significant differences between groups in mean EQ-5D index values, EQ VAS scores, and ASCOT-Carer scores at p < 0.01 level

### Construct validity

Two established measures were used to evaluate construct validity (specifically convergent validity) of LTCQ-Carer: EQ-5D-5L with EQ VAS, and ASCOT-Carer. The patient version of LTCQ correlated very strongly and positively with EQ-5D (*r*_s_ = 0.82, *p* < 0.001 (index value); *r*_s_ = 0.79, *p* < 0.001 (EQ VAS)) [25], and we hypothesized that LTCQ-Carer’s general construct of ‘living well while providing care and support for someone with LTCs’ would also correlate positively and at least moderately with EQ-5D’s general construct of ‘health-related quality of life’. A stronger association was predicted between LTCQ-Carer and ASCOT-Carer, as there was more direct overlap between concepts represented by items across these two scales. For each set of measures, statistically significant associations of scores were observed in the predicted directions, supporting construct validity: *r*_s_ = 0.52, *p* < 0.001 (LTCQ-Carer and EQ-5D-5L index value), *r*_s_ = 0.61, *p* < 0.001 (LTCQ-Carer and EQ VAS), *r*_s_ = 0.85, *p* < 0.001 (LTCQ-Carer and ASCOT-Carer). Results of ANOVA analysis to test known-groups validity are reported in Table 3.

### Responsiveness

Table 4 shows mean LTCQ-Carer scores for respondents to the four-month follow-up survey (*n* = 51), grouped by reported change in health since the first survey. The sample size, particularly for those reporting improved health (*n* = 4), was too small to undertake comparative sub-group analysis. No statistically significant change was observed for mean LTCQ-Carer scores across the two time points. This might be a reflection of most of the sample reporting no change in health status over the four months, but we cannot interpret this result with confidence.

**Table 4:** Change in LTCQ-Carer score and global change in health status over four months, Survey 2

Health at Survey 2 compared to Survey 1	N	Mean LTCQ-Carer score—Survey 2 (SD, SE)	Mean LTCQ score—Survey 1 (SD, SE)	Mean change in LTCQ score (SD, SE)
Better than 4 months ago	4	73.5 (9.0, 4.5)	70.5 (21.2, 10.6)	2.97 (15.9, 7.9)^a^
About the same as 4 months ago	33	80.1 (14.2, 2.5)	80.6 (14.9, 2.6)	− 0.47 (7.1, 1.2)^a^
Worse than 4 months ago	14	53.8 (9.7, 2.6)	53.2 (11.6, 3.2)	0.68 (9.6, 2.7)^a^
Total	51	72.0 (17.5, 2.5)	72.6 (18.7, 2.6)	0.11 (8.5, 1.2)^a^

*SD* standard deviation, *SE* standard error of the mean

^a^Following paired-samples t-tests to compare mean LTCQ-Carer scores between Survey 1 and Survey 2, none of the observed changes in mean scores were statistically significant, either for the follow-up sample as a whole (*n* = 51) or for any of the sub-groups based on self-reported global change in health status between surveys

### Potential for use within the memory services clinical pathway

Ongoing feedback from memory clinic staff indicated that LTCQ-Carer responses provided valuable information that might not otherwise have been captured at the point of the cared-for patient’s initial diagnosis. On being given the study pack some participants opted to complete Survey 1 on site before the end of the appointment, which provided an unanticipated opportunity for carers to share their responses directly with health professionals. One senior memory clinic nurse who experienced conversations arising from the completed questionnaire stated: “I found the questionnaire a useful clinical tool as it elicited more information, particularly from carers, who seemed to feel able to disclose more in response to the questionnaire, even though the topics were raised during the assessment. The disclosures (often distress) meant that their issues could be addressed through a more formal carer assessment on-line or by [local carer support organisation]. Perhaps there could be a link with the Carer’s assessment [by the Local Authority responsible for social care support]?” Feedback from memory clinic staff thus suggested that completion of LTCQ-Carer during the patient’s assessment could prompt earlier discussion of need or distress among family carers, potentially identifying and acting on such needs before the carer reaches a crisis point.

## Discussion

In this study, we demonstrated that LTCQ-Carer is a valid means of capturing quality-of-life outcomes for informal (family) caregivers supporting people recently diagnosed with MCI or dementia. We established the principle of administering a person-centred outcome measure to carers at the point of the patient’s diagnosis, provided that the clinical workflow allows some opportunity for health professionals to engage independently with carers. This was the case in the memory clinic setting for which LTCQ-Carer was developed, as the appointment structure included time for a private discussion between the carer and one health professional while a second health professional administered memory tests with the patient. Taking the earliest opportunities to ask carers about their own health and well-being (and not just their skills as carers) aligns with the Make Every Contact Count (MECC) approach within England’s health and care services, which aims to take each clinical encounter as an opportunity to ‘engage [individuals] in conversations about their health at scale across organisations and populations’ [40].

Study results support our hypothesis that aspects of ‘living well’ that are relevant for people living with long-term health conditions [1, 29] are also relevant for the people who support them. While patients and carers assessed their own quality of life with reference to different influences (health conditions versus experiences of providing care), broad domains such as a sense of control, feeling safe, and ability to do meaningful activities were endorsed as important by both groups. The similarity of content between LTCQ and LTCQ-Carer could prove useful for understanding the impacts of LTCs (direct impacts for patients, indirect impacts for carers) at the household/family level [32], rather than only considering impacts on individuals in isolation. Furthermore, we demonstrated the extent to which carers are patients too; we observed a high burden of LTCs in the carers themselves, which needs to be accounted for in monitoring carers’ overall quality of life.

The conceptual breadth of LTCQ-Carer, bridging health- and social care-related quality of life, fills a gap in available assessment tools that consider these constructs separately. When planning for this project, the most promising available measure was ASCOT-Carer, which was validated through robust measures [24] and performs well in comparison to other preference-based measures used for economic evaluations [41]. However as noted in a recent review [42], its measured construct ‘social care-related quality of life’ is potentially too narrow for capturing all outcomes of interest to health professionals supporting informal carers, or to carers themselves. Recently developed carer measures that are specific to dementia, such as C-DEMQOL [43] and SIDECAR [44] may similarly be well suited for some applications but may be limited for comparability with other groups of carers. Manthorpe and Bowling [19] conclude that ‘generic versus condition-specific QoL measures for carers require assessment but there is also the complexity that most people with dementia have multiple long-term disabilities and/or impairments and that these are interrelated’. Page et al. [45] concurred, noting considerable overlap of domains across carer measures for different neurological conditions and recommending that the feasibility of using a shorter single measure for carers be explored. LTCQ-Carer could potentially be used for this purpose, with further testing to confirm validity in other carer populations.

Nonetheless our results must be interpreted cautiously owing to study limitations. A theoretical limitation was that a conceptual framework for LTCQ-Carer was not developed de novo from qualitative interviews with carers; instead content was adapted from an existing validated measure (LTCQ). This choice reflects the logical extension of the study from its original purpose (validation of LTCQ among memory clinic patients), where clinical partners sought to assess carer quality of life using the same domains as LTCQ. Synergy between the two measures potentially increases familiarity among professional users (who might administer LTCQ to patients at the same time as administering LTCQ-Carer to family members), and might allow some degree of comparability across patient and carer populations. But in the absence of initial exploratory interviews with carers, some potentially important domains might not have been explicitly tested during the cognitive interview phase, although interviewees were prompted generally to consider if any important content was missing from the questionnaire.

We are limited in generalising the applicability of the measure to all informal carers, since LTCQ-Carer was developed and tested specifically with carers of people with MCI/dementia. LTCQ-Carer should be tested in other carer/patient populations whose experiences might differ from our relatively older study sample, for example parents looking after children with life-long illness. Carer experiences and outcomes might also change over time or vary by the patient’s condition (e.g. heart failure patients whose support needs might be more intermittent than for dementia patients). There is also potential variation according to illness severity; we were unable to account for this in our study since the research team did not have access to clinical data on the level of the cared-for person’s cognitive impairment. The ability of LTCQ-Carer to detect these potential variations should be explored in future studies. The small sample size, while allowing for adequate quality in the psychometric analyses undertaken [33], mean that results for structural validity and responsiveness are only suggestive at this stage. A larger study with a wider range of carers and over a longer period of follow-up is needed to confirm (or re-interpret) the results from this pilot validation survey, in particular to address the general lack of evidence on responsiveness to change in carers’ health and wellbeing over time [42].

## Conclusions

The need to support informal carers to live well is firmly established in national and international policy. LTCQ-Carer is a valuable addition to the emerging suite of high-quality measures for robust assessment of carers’ own quality of life. LTCQ-Carer is a valid means of capturing outcomes of importance for carers of people living with cognitive impairment including dementia. It should be further test among larger samples and other carer groups to potentially fill a gap in generic measures of carers’ quality of life, across the full spectrum of long-term conditions. This study has demonstrated the potential of LTCQ-Carer as a person-centred tool for building an evidence base around carer support, which is a crucial element of understanding the full impact of long-term health conditions at population level.

## Data Availability

Data are held by the research team at the University of Oxford, as stated on the Participant Information Sheet: ‘We will keep research data (questionnaire responses) for 10 years and then they will be destroyed. All data use is strictly within the terms of the Data Protection Act (DPA 1998). Only the research team will have regular access to research data, and any data that is shared with others will have names and any other personal information removed so that you cannot be identified from it. Any information collected as part of the study may also be seen by responsible persons from the study sponsor (University of Oxford) or regulatory authorities, but only where it is required for monitoring research. Data will not be shared outside the research team for any other purposes.’
